# Urgent Need for Field Surveys of Coronaviruses in Southeast Asia to Understand the SARS-CoV-2 Phylogeny and Risk Assessment for Future Outbreaks †

**DOI:** 10.3390/biom11030398

**Published:** 2021-03-09

**Authors:** Murat Seyran, Sk. Sarif Hassan, Vladimir N. Uversky, Pabitra Pal Choudhury, Bruce D. Uhal, Kenneth Lundstrom, Diksha Attrish, Nima Rezaei, Alaa A. A. Aljabali, Shinjini Ghosh, Damiano Pizzol, Parise Adadi, Tarek Mohamed Abd El-Aziz, Ramesh Kandimalla, Murtaza M. Tambuwala, Amos Lal, Gajendra Kumar Azad, Samendra P. Sherchan, Wagner Baetas-da-Cruz, Giorgio Palù, Adam M. Brufsky

**Affiliations:** 1 Doctoral Studies in Natural and Technical Sciences (SPL 44), University of Vienna, Währinger Straße, A-1090 Vienna, Austria; a11851761@unet.univie.ac.at; 2 Department of Mathematics, Pingla Thana Mahavidyalaya, Maligram, Paschim Medinipur 721140, West Bengal, India; sarimif@gmail.com; 3 Department of Molecular Medicine, Morsani College of Medicine, University of South Florida, Tampa, FL 33612, USA; 4 Applied Statistics Unit, Indian Statistical Institute, Kolkata 700108, West Bengal, India; pabitrapalchoudhury@gmail.com; 5 Department of Physiology, Michigan State University, East Lansing, MI 48824, USA; bduhal@gmail.com; 6 PanTherapeutics, Rte de Lavaux 49, CH1095 Lutry, Switzerland; 7 Dr. B R Ambedkar Center for Biomedical Research (ACBR), University of Delhi (North Camps), Delhi-110007, India; dikshaattrish@gmail.com; 8 Research Center for Immunodeficiencies, Pediatrics Center of Excellence, Children’s Medical Center, Tehran, University of Medical Sciences, Tehran 1419733151, Iran; rezaei_nima@yahoo.com; 9 Network of Immunity in Infection, Malignancy and Autoimmunity (NIIMA), Universal Scientific Education and Research Network (USERN), Tehran 1419733151, Iran; 10 Department of Pharmaceutics and Pharmaceutical Technology, Yarmouk University-Faculty of Pharmacy, Irbid 566, Jordan; alaaj@yu.edu.jo; 11 Department of Biophysics, Molecular Biology and Bioinformatics, University of Calcutta, Kolkata 700009, West Bengal, India; shinjinighosh2014@gmail.com; 12 Italian Agency for Development Cooperation—Khartoum, Sudan Street 33, Al Amarat 13374, Sudan; damianopizzol8@gmail.com; 13 Department of Food Science, University of Otago, Dunedin 9054, New Zealand; pariseadadi@gmail.com; 14 Department of Cellular and Integrative Physiology, University of Texas Health Science Center at San Antonio, 7703 Floyd Curl Dr, San Antonio, TX 78229-3900, USA; mohamedt1@uthscsa.edu; 15 Zoology Department, Faculty of Science, Minia University, El-Minia 61519, Egypt; 16 CSIR-Indian Institute of Chemical Technology Uppal Road, Tarnaka, Hyderabad 500007, Telangana State, India; ramesh.kandimalla@gmail.com; 17 School of Pharmacy and Pharmaceutical Science, Ulster University, Coleraine BT52 1SA, Northern Ireland, UK; m.tambuwala@ulster.ac.uk; 18 Division of Pulmonary and Critical Care Medicine, Mayo Clinic, Rochester, MN 55905, USA; manavamos@gmail.com; 19 Department of Zoology, Patna University, Patna 800005, Bihar, India; gkazad@patnauniversity.ac.in; 20 Department of Environmental Health Sciences, Tulane University, New Orleans, LA 70112, USA; sshercha@tulane.edu; 21 Translational Laboratory in Molecular Physiology, Centre for Experimental Surgery, College of Medicine, Federal University of Rio de Janeiro (UFRJ), Rio de Janeiro 21941901, Brazil; wagner.baetas@gmail.com; 22 Department of Molecular Medicine, University of Padova, Via Gabelli 63, 35121 Padova, Italy; 23 UPMC Hillman Cancer Center, Department of Medicine, Division of Hematology/Oncology, University of Pittsburgh School of Medicine, Pittsburgh, PA 15213, USA; brufskyam@upmc.edu

Phylogenetic analysis of severe acute respiratory syndrome coronavirus 2 (SARS-CoV-2) is focused on a single isolate of bat coronaviruses (bat CoVs) which does not adequately represent genetically related coronaviruses (CoVs). The unique bat CoV RaTG13 is the only identified sequence genetically associated with SARS-CoV-2. Data scarcity of bat CoV sequences raises concerns over several fundamental experimental and biostatistical aspects, e.g., repeatability of sequences and intraspecies variations in critical gene regions, such as the receptor-binding domain of the spike protein. The Sunda pangolin has been proposed as the intermediate host and source of SARS-CoV-2, but no pangolin CoV isolates have been reported in its habitats in Southeast Asia. Most pangolin CoVs were isolated from pangolins captured during illegal animal trafficking, raising questions about such isolates’ reliability and quality. Problems with pangolin CoV sampling are also related to the substandard quality of deposited sequences. There is an urgent need for field surveys of bat CoVs and possible intermediate hosts, such as pangolins, ferrets, and civets, in Southeast Asia to investigate the genomic source of SARS-CoV-2 and assess possible future risks for new outbreaks.

SARS-CoV-2 is the causative agent of the coronavirus disease 2019 (COVID-19) pandemic in which the first cases were reported in Wuhan, Hubei Province, China. SARS-CoV-2, a member of *Betacoronavirus* and subgenus *Sarbecovirus*, is phylogenetically related to bat coronaviruses (bat CoVs) RaTG13 (detected in Pu’er City in 2013) and RmYN02 (detected in Xishuangbanna City in 2019) and were detected approximately 2000 km from Wuhan [1,2]. *Betacoronavirus* and its genetic reservoir bat species, such as the intermediate horseshoe bat *Rhinolophus affinis*, inhabit Southeast Asia [1,3,4]. It has been hypothesized that pangolin CoVs may have originated from cross-species transmission in bats [5]. To date, no additional bat CoVs RaTG13 or RmYN02 isolates and no pangolin CoV isolates originating from healthy or sick pangolins in their natural habitats in Southeast Asian countries have been sequenced (Figure 1). Therefore, here, we seek to draw the attention of relevant institutions toward the need to obtain CoV isolates from potential hosts, such as healthy or sick pangolins, in their natural habitats to match the existing sequences.

Based on genomic and sequence data submitted on pangolin CoVs in early 2020, pangolins were proposed as the intermediate host for SARS-CoV-2 [5,6,7,8]. Recent genomic analysis suggests the possible recombination of bat CoVs and pangolin CoVs, which occurred at least twice, leading to the creation of SARS-CoV- 2 [9]. Furthermore, pangolin cells lacking the interferon-induced helicase C domain 1 (IFIH1) and Z-DNA-binding protein (ZBP1) have been postulated to contribute to the switch from resistance to tolerance of CoV infections [10]. Pangolins such as the giant pangolin, *Smutsia gigantea*, inhabit the same caves as different bat species such as *Hipposideridae* sp., *Emballonuridae* sp., and *Miniopterus* sp. in Gabon, which could also be the case in Southeast Asia [11].

Illegal animal trafficking of infected pangolins has been postulated as the possible transmission route of SARS-CoV-2 to its epicenter at Wuhan’s wet seafood animal market [6,7,8]. The Sunda pangolin (Manis javanica) is native to Southeast Asian countries, such as Cambodia, Myanmar, Thailand, Laos, Vietnam, and a small area in Yunnan, China, where the potential source of the SARS-CoV-2 intermediate horseshoe bat is also present [1,3,12]. Under these conditions, the Sunda pangolin would serve as the intermediate host, and SARS-CoV-2 would have emerged through spillover from pangolins to humans once (scenario I) or several times (scenario II) [5]. It is also possible that SARS-CoV-2 and pangolin CoVs would have originated independently through the cross-transmission of bat species (scenario III) [5]. Interestingly, pangolin CoVs have also been detected approximately 1000 km from Wuhan (Guangxi Province, 2017, and Guangdong Province, 2019) [6,7,8].

SARS-CoV-2 has been suggested to diverge from the lineage of bat CoV Ratg13 in 1969, with the highest posterior density interval of 95% for the years 1930 to 2000 [13]. To date, no isolates other than bat CoVs RaTG13 and RmYN02 have been discovered, resulting in incomplete phylogenetic analyses, such as the selection of midpoint rooting, limited taxon sampling, and inappropriate emphasis on one single element such as ACE2 [14]. The intraspecies phylogeny of the SARS-CoV-2 clinical isolate has been criticized, and it has been requested to utilize larger datasets and sample variability, which are also required for the determination of the interspecies SARS-CoV-2 phylogeny with bat CoVs, e.g., RaTG13 [14,15]. Most phylogenetic studies ignore the necessity of sufficient intraspecies sample sizes and the assessment of intraspecies genomic variations [16].

The findings from comparative phylogenetic analyses can be influenced by intraspecies sample sizes [16], which might be the case for SARS-CoV-2. If the intraspecific genomic variation is high, a large dataset is necessary due to potential heterogeneity [16]. For instance, the pangolin CoVs and SARS-CoV-2 spike (S) protein receptor-binding domains (RBDs) are almost identical [8]. However, if the intraspecies variability in the S protein RBD of pangolin CoVs is high, the assessment of the genetic relatedness to SARS-CoV-2 is unreliable. This also applies to the phylogenetic analysis of SARS-CoV-2 with bat CoV species.

However, if the S protein RBD in the SARS-CoV-2 clade can be considered as conserved, the genomic relatedness to RmYN02 is irrelevant since their RBD homology is low [2]. Similarly, based on a single sequence of RmYN02, the furin protease cleavage insert of SARS-CoV-2 was proposed [2,17]. If S1/S2 in the S protein of RmYN02 is a variable and a discrete trait of this viral species, these genetic conclusions are irrelevant [2]. 

Reproducibility, seasonality, population differences, and measurement errors of sequences of bat CoVs and pangolin CoVs have not been validated [2,6,7,8,13]. Additionally, there could be sequencing errors due to several factors such as poor sample quality, improper handling, secondary PCR enrichment, and low-quality measurements [16,18]. The RaTG13 strain was isolated in 2013, but its complete genomic sequence (GenBank ID MN996532) was submitted after the emergence of SARS-CoV-2 in 2020 [19]. Additionally, the RNA-dependent RNA polymerase (RdRp) gene of RaTG13 is identical to that of another bat CoV sequence BtCoV/4991 (GenBank ID KP876546) submitted in 2015 [19]. Strikingly, NCBI KRONA analysis of the RaTG13 sequence suggested the possibility of DNA contamination, and the sequence was considered a fossil record [7,20]. For example, the analysis relied on the pangolin CoV sequence MP789 on GISAID (https://www.gisaid.org/EPI_ISL_412860hCoV-19/pangolin/China/MP789/2019, accessed on 14 September 2020). However, the database commented on sequences with long stretches of unreadable bases in nucleic acid sequences annotated as NNNs (about 7%) and missing NSP14. Pangolin CoV MP789 was collected from the lungs of dead pangolins [7]. The sequencing was completed with gap-filling PCR, and the filled version was deposited in GenBank (MT121216.1) [7]. Although gap filling is a standard protocol, this indicates the need for better sample and sequence quality for pangolin CoVs. The gap filling had quality issues, missing data, and unexpected reads possibly due to contamination by other viruses, including SARS-CoV-2-related viruses, and mitochondrial genes from pangolins (NC_026781), humans (NC_012920), tigers (NC_010642), and mice (NC_005089) were identified [21]. Similarly, pangolin CoV isolate P3B, collected from Sunda pangolin blood, possesses long sequence stretches of unreadable NNNs (8% of the sequence), and NSP3, NSP2, NSP6, NSP4, NSP15, and NSP8 were missing in this sample. The sequence of P3B was characterized using a gap-filling protocol, but the completed sequence has not been deposited in GenBank [6].

Based on three single isolates of bat CoVs (RaTG13, ZC45, and ZXC21) and gap-filled pangolin CoV (2017 and 2019 isolates) sequences, it was postulated that SARS-CoV-2 diversified as a species 70 years ago and remained undetected [12]. These analyzed sequences are an insufficient representation of all genetically related sarbecoviruses to SARS-CoV-2 in Southeast Asia [1,3,13]. This raises the question of how the wild-type SARS-CoV-2 has existed for 70 years without infecting humans. Many people involved in the hunting and trade of pangolins should have been exposed to the SARS-CoV-2 ancestor before the Wuhan outbreak [12]. For instance, approximately 83 bat species are consumed in 33 different countries, including 13 species in New Guinea and 14 species in the Philippines [22]. Considering the high mobility of bat species consumed by humans as bushmeats, there is a high possibility of direct contact of humans with SARS-CoV-2 [1]. Additionally, the higher risk of CoV transmission to other animals and humans is not due to meat consumption but contamination of water sources, since CoVs exist in bat feces [23]. For example, bats contaminated wells and ponds with MERS-CoV, which infected camels, leading to humans [23].

However, the reason why SARS-CoV-2 remained dormant for 70 years could be related to its different pathology in bats. CoVs like RaTG13 infect the bat gastrointestinal system, since they have been detected in the feces and intestines of bats [23]. Bats cope well with CoVs, eliciting robust amounts of antibody responses [23]. In a study conducted in Yunnan Province on feces from the Chinese rufous horseshoe bat *Rhinolophus sinicus*, CoV was detected only in 12 of the 164 samples [4]. Therefore, bat-mediated host tropism events could have lesser proximity than expected due to the low frequency of bat CoV infection. Moreover, in a study conducted in Malaysia from 2009 to 2019 on 334 Sunda pangolins, none of the samples were positive for CoVs as verified by PCR [24].

The SARS-CoV-2 host range has been evaluated based on the interaction with the ACE2 entry receptor [25,26,27]. Despite residues 24, 30, 34, 38, 82, and 354 being common in both human and pangolin ACE2 protein, the binding affinity is much lower for pangolin ACE2, indicating that pangolins are not an intermediate host for the COVID-19 pandemic [26,27]. Moreover, SARS-CoV-2 can use other entry pathways such as C-type lectin receptors (CLRs) and neuropilin [28]. Therefore, SARS-CoV-2 might be capable of infecting host species with an incompatible ACE2 protein structure, such as mice and chickens [25,27]. Moreover, the presence of SARS-CoV-2 has been serologically confirmed in minks (*Neovison vison*), cats (*Felis catus*), ferrets (*Mustela putorius furo*), Chinese tree shrews (*Tupaia belangeri chinensis*), rhesus macaques (*Macaca mulatta*), domestic pigs (*Sus domesticus*), cynomolgus or crab-eating macaques (*Macaca fascicularis*), racoon dogs (*Nyctereutes procyonoides*), common marmosets (*Callithrix jacchus*), hamsters (*Mesocricetus auratus*), African green or vervet monkeys (*Chlorocebus aethiops*), dogs (*Canis familiaris*), fruit bats (*Rousettus aegyptiacus*), tigers (*Panthera tigris*), and lions (*Panthera leo*), along with mild or moderate infections in some species [29,30,31,32,33,34,35,36,37]. Most cases could be traced back to human-to-animal transmission, but in some cases, fatal infections with an animal-to-animal transmission in minks and cats were detected, which indicates the highly contagious nature of SARS-CoV-2 [38,39,40,41]. Even mink-to-human back-infections have been described [39]. Given the pathological potential of SARS-CoV-2, a field survey to acquire large complete-genome samples of CoVs for various inter- and intraspecies analyses is essential to investigate the zoonotic origin of SARS-CoV-2 or to discover genetic diversity/unity among various CoVs and especially sarbecoviruses in Southeast Asia [1,2]. Furthermore, it will enrich information in the existing bat CoV and pangolin CoV species pool, leading to the discovery of CoVs in other potential hosts and expanding intraspecies sampling.

The foundation of humankind is vulnerable in the context of the pathological capacity of CoVs, especially SARS-CoV-2, a member of the *Sarbecovirus* subgenus that is responsible for the ongoing COVID-19 pandemic [42]. Expanded intra- and interspecies sampling for bat CoV RaTG13 and pangolin CoVs in Southeast Asia seems to be a reasonable scientific step critical for reliably elucidating the phylogenetic composition of the *Sarbecovirus* subgenus and its members, which are genetically related to SARS-CoV-2 [1,2]. Multiple isolates from different locations, times, and hosts of bats, pangolins, and other potential populations of CoVs are essential for improving understanding of the adaptations, mutations, and recombination patterns of genetically related clades of SARS-CoV-2. Furthermore, if SARS-CoV-2 is unrelated to RaTG13, its true bat CoV ancestor is still awaiting to be discovered. Therefore, there is an urgent need for CoV surveys on bats and possible intermediate hosts in Southeast Asia to reliably investigate the SARS-CoV-2 ancestry and identify genetically related sarbecoviruses with a similar pathological capacity to prevent future CoV outbreaks [42].

## Figures and Tables

**Figure 1:**
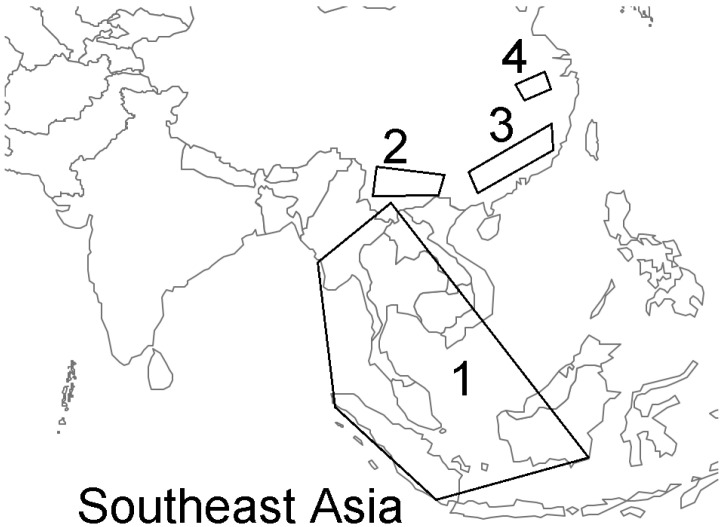
Zone 1 is the area where Sunda pangolins are distributed in Southeast Asia and the potential source area of severe acute respiratory syndrome coronavirus 2 (SARS-CoV-2) that has never been screened for genetically related clades of coronaviruses (CoVs). Zone 2 is the location where bat coronaviruses (bat CoVs) RaTG13 and RmYN02 were isolated in Yunnan, China. Zone 3 is where Sunda pangolin CoVs were isolated from dead pangolins in Guangxi and Guangdong, China. Zone 4 is the location where SARS-CoV-2 was first reported from its epicenter in Wuhan [3].
